# Nitrous oxide (N_2_O) and subsequent open-label SSRI treatment of adolescents with depression (NOTAD): study protocol for a randomised controlled trial

**DOI:** 10.1186/s13063-017-2342-4

**Published:** 2017-12-22

**Authors:** Richard M. Stewart, Janice W. Y. Wong, Kevin C. Runions, Pradeep Rao, Julia K. Moore, Simon R. Davies, Britta S. von Ungern-Sternberg, David Sommerfield, Florian Daniel Zepf

**Affiliations:** 1 Centre & Discipline of Child and Adolescent Psychiatry, Psychosomatics and Psychotherapy, School of Medicine, Division of Paediatrics and Child Health & Division of Psychiatry and Clinical Neurosciences, The University of Western Australia, 35 Stirling Highway (M561), Crawley, WA 6009 Perth, Australia; 2 Telethon Kids Institute, Perth, Australia; 3 Specialised Child and Adolescent Mental Health Services (CAMHS), Department of Health, Perth, Australia; 4 Community Child and Adolescent Mental Health Services (CAMHS), Department of Health, Perth, Australia; 5 Acute Child and Adolescent Mental Health Services (CAMHS), Department of Health, Perth, Australia; 6 Department of Anaesthesia and Pain Management, Princess Margaret Hospital for Children, Perth, Australia; 7 School of Medicine and Pharmacology, The University of Western Australia, Perth, Australia

**Keywords:** Depression, Children, Adolescents, Nitrous oxide, NMDA, Psychopharmacology, Mood disorders, Neuroscience

## Abstract

**Background:**

The first line of pharmacological treatment for severe depressive disorders in young people is selective serotonin reuptake inhibitors (SSRIs). However, beneficial clinical effects are rarely observed before several weeks into treatment. Nitrous oxide (N_2_O) has a long-standing safety record for pain relief and has been used in adults and young people. In adults with severe treatment-resistant depression, a single dose of N_2_O had significant antidepressant effects, with maximum antidepressant effects observed 24 h after administration. However, the antidepressant effects of N_2_O have never been investigated in adolescents with a confirmed diagnosis of depression in a prospective trial. The aims of this study are to (1) investigate whether a single inhaled N_2_O administration leads to antidepressant effects in adolescents with depression at 24 h, (2) determine whether combined N_2_O and SSRI administration (commenced after N_2_O intervention) provides a clinically significant improvement in mood over and above the benefits from SSRI administration alone, and, (3) investigate whether the effect seen following N_2_O administration can be used as a predictor of SSRI treatment response.

**Methods/design:**

In this study, we will use a single-blind, randomised, placebo-controlled design. Patients aged between 12 and 17 years with major depressive disorder will be recruited. This study will consist of two phases: phase A and phase B. During phase A, participants will be randomised to receive either inhaled N_2_O or placebo (air) for 1 h. In phase B, participants will receive open-label pharmacological treatment with the SSRI fluoxetine and will be followed over a 12-week period. Participants will undertake mood assessments at 2 and 24 h after N_2_O or placebo administration (phase A) and weekly during the 12-week follow up in phase B.

**Discussion:**

We expect an antidepressant effect from a single dose of inhaled N_2_O compared with placebo at 24 h after administration. Additionally, we expect that subjects treated with N_2_O will also show greater improvements than the placebo group after 6 and 12 weeks into fluoxetine treatment because of potential additive antidepressant effects. Such findings would be of clinical importance because currently children and adolescents often do not experience any symptom alleviation for several weeks following the initiation of SSRIs.

**Trial registration:**

Australian and New Zealand Clinical Trials Registry, ACTRN12616001568404. Registered on 14 November 2016.

**Electronic supplementary material:**

The online version of this article (doi:10.1186/s13063-017-2342-4) contains supplementary material, which is available to authorized users.

## Background

One in 16 young Australians is currently experiencing depressive symptoms, which underlines the significant prevalence of depressive disorders in minors [1]. Approximately 6.3% of Australians aged between 16 and 24 years have experienced an affective disorder in the last 12 months [1]. Selective serotonin reuptake inhibitors (SSRIs) are frequently used for the pharmacological treatment of depressive disorders. SSRIs are amongst the most frequently prescribed antidepressants and are known to increase the availability of the neurotransmitter serotonin (5-HT) in the brain, which is thought to contribute to their antidepressant effects. However, the complete mechanism of action of SSRIs is still not fully understood. In particular, the question why antidepressant effects are often observed only after a delay of several weeks into treatment, despite the fact that single doses of SSRI administration can lead to significant changes in activation of brain regions that are known to be associated with mood and emotion regulation (i.e., the amygdala, hippocampus and prefrontal cortex) [2]. The described delay in the onset of antidepressant effects after SSRI administration can put young patients at risk for exacerbation of low mood and other associated depressive symptoms, such as self-harm or suicide. Thus, two major child and adolescent health issues are highlighted and must be urgently addressed: first, whether there is a treatment strategy that can bridge the gap between the initiation of SSRIs and observed (possibly also additional) clinical benefits, and second, the need to investigate the predictors of response to SSRI treatment, which in turn could save significant time allocated to treatments with little or no effect.

Strong biological and recent clinical evidence has suggested that nitrous oxide (N_2_O) has significant antidepressant effects in adults, in particular in patients with treatment-resistant depression (TRD) [3]. The underlying mechanism of action of N_2_O is thought to be through the *N*-methyl-d-aspartate (NMDA) receptor, because N_2_O serves as an antagonist of this particular receptor [4–6]. Numerous animal studies and human trials have investigated the use of NMDA antagonists in targeting behaviours or symptoms that may be associated with depression. For example, in murine models, the administration of ketamine, which, like N_2_O, is an NMDA antagonist and anaesthetic, showed a reduction in depression-like behaviour, such as increased mobility in the forced-swim test, shorter latencies in the novelty-suppressed feeding test and a reduction in learnt helplessness [7, 8]. In this context, authors of a recent systematic review of studies investigating the use of ketamine to treat TRD in humans suggested it to be associated with a rapid onset of antidepressant effects [9]. Similarly, in a trial where inhaled N_2_O was administered in adults with TRD, Nagele et al. [3], found a significant improvement in depressive symptoms as rated by the 21-item Hamilton Depression Rating Scale (HDRS) at 2 and 24 h and 1 week (in a subset of participants) following administration. These findings are significant because in the underlying neurobiology of depression, the NMDA receptor is thought to play a decisive role [8, 10, 11]. NMDA antagonists, such as N_2_O and ketamine, work by blocking the NMDA receptor, which inhibits a further inhibiting interneuron (GABAergic), resulting in a release of glutamate. Whilst the blocking of the NMDA receptor leads to this sequential effect which impacts three neurons, it has been hypothesised that the blocking of the NMDA receptor leads primarily to an almost immediate antidepressant effect [12–15]. The mechanism of action of SSRIs differs from that of the NMDA antagonists, because it may take a period of weeks following administration before the desensitisation of central nervous system 5-HT receptors occurs, which has been hypothesised to lead to an increase of 5-HT within the synaptic cleft (i.e., antidepressant effect). Therefore, the use of N_2_O, a safe inhalational anaesthetic, has the potential to be a novel, fast-acting and non-intrusive treatment strategy for patients with severe depressive disorders while awaiting onset of SSRI antidepressant activity. Furthermore, in the hospital setting, N_2_O has been used for several decades with an excellent record with regard to safety and effectiveness.

Whilst the described mechanisms of action in depression of NMDA antagonists and SSRIs are not fully understood, animal models have shown that the administration of NMDA antagonists is associated with changes in specific brain areas which are also related to mood, such as the hippocampus and the prefrontal cortex [16]. Most notably, such areas are also highly 5-HT-modulated areas of the brain [4, 17]. Given the significant overlap in neurocircuits, an increase in brain function in terms of improved top-down control in mood-related neurocircuits may be used to explain any possible additive treatment effects of the administration of both N_2_O and SSRIs (e.g., fluoxetine), as well as for investigating N_2_O as a potential predictor of SSRI response. Whilst the significant antidepressant effects of N_2_O in a cohort of adults with TRD has been shown [3], such research questions have never been investigated in minors.

### Study objectives and hypotheses

The objectives and hypotheses of the present study are threefold:

1. *To investigate the antidepressant effects of 1-h administration of inhaled N*_*2*_ *O at 24 h post-administration compared with placebo (phase A of the study)*: We hypothesise that a single inhaled administration of N_2_O will lead to a reduction of depressive symptoms in young people with depression compared with a placebo group. Depressive symptoms will be measured through the use of the Beck Depression Inventory (BDI), the HDRS, and the Children’s Depression Rating Scale–Revised (CDRS-R).
2. *To explore whether a single administration of N*_*2*_ *O provides any additional improvement in mood when followed by open-label SSRI administration (phase B of the study) over and above the benefits from SSRI administration alone*: We hypothesise that participants treated with N_2_O will show greater improvements in mood (as indicated by change in BDI, HDRS and CDRS-R scores), owing to possible additive effects and possibly faster symptom remission, after 6 and 12 weeks into open-label fluoxetine treatment (phase B of the study) compared with the placebo group.
3. *To explore whether antidepressant effects (as indicated by changes in BDI, HDRS and CDRS-R scores) associated with a single administration of N*_*2*_ *O can act as a predictor of SSRI response*: Given the potential overlap in neurocircuits that are associated with the administration of NMDA antagonists and 5-HT, we hypothesise that the use of N_2_O may predict response to the use of the SSRI fluoxetine as a pharmacological treatment.

## Methods/design

### Trial design

The present study will employ a randomised, placebo-controlled, blinded clinical pilot trial design for phase A (N_2_O or placebo), followed by an open-label design using fluoxetine administration in phase B of the study.

### Setting of the study

The present study will be conducted at Princess Margaret Hospital (PMH), Perth, Australia, and is expected to be transferred to the new Perth Children’s Hospital once opened.

### Consent

Written informed consent will be obtained from participants and their parents/guardians by the study psychiatrists at a baseline session. Once consent has been obtained, the study team will arrange thyroid function tests and electrocardiography for each participant to ensure that fluoxetine is a suitable medication for the individual participant to take during phase B of the study.

### Subjects and eligibility criteria

In this study, we aim to recruit a total of 30 participants. There are three key inclusion criteria. First, participants must have a confirmed diagnosis of moderate to severe major depressive disorder according to the International Classification of Diseases, Tenth Revision, or the *Diagnostic and Statistical Manual of Mental Disorders, Fifth Edition*, criteria, with the diagnosis to be confirmed through the administration of the Kiddie Schedule for Affective Disorders and Schizophrenia (K-SADS) [18] as well as a full psychiatric assessment. Second, participants must be aged between 12 and 17 years. Third, participants and their parents/guardians must agree to start fluoxetine as treatment in phase B of the study. Exclusion criteria include any active suicidal ideation or plans, current use of other psychiatric medications at study entry, IQ < 85 (screened using the Wechsler Abbreviated Scale of Intelligence [WASI-II] [19]), active or past diagnosis of an eating disorder, current or recent (i.e., past 12 months) abuse or dependence on alcohol or illicit substances, current pregnancy in female participants, significant chronic medical or neurological disorder, history of bipolar disorder, schizophrenia, schizoaffective disorder, obsessive compulsive disorder or personality disorders, presence of an acute medical illness that could interfere with study participation, active psychotic symptoms, administration of NMDA receptor antagonists during the preceding 3 months, ongoing or past treatment with electroconvulsive therapy, contraindications against the use of N_2_O and contraindications against the use of fluoxetine.

### Intervention

**Fig. 1:**
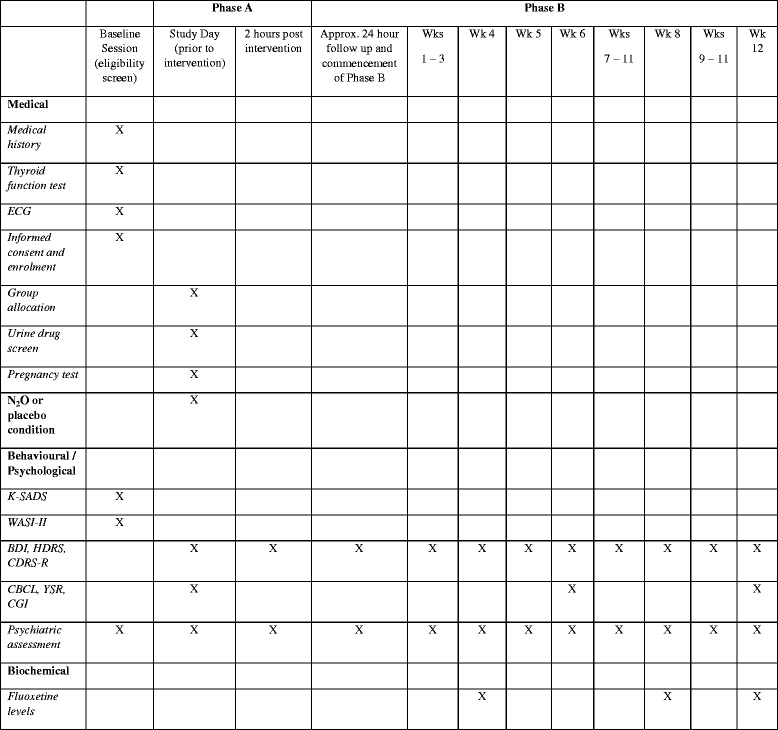
An illustration of the medical, biochemical, and psychological assessments that will be conducted during the Nitrous oxide and subsequent open-label SSRI treatment of adolescents with depression (NOTAD) study. *ECG* Electrocardiography, *BDI* Beck Depression Inventory, *CBCL* Child Behaviour Checklist, *CDRS-R* Children’s Depression Rating Scale–Revised, *CGI* Clinical Global Impression, *HDRS* Hamilton Depression Rating Scale, *K-SADS* Kiddie Schedule for Affective Disorders and Schizophrenia, *N*_*2*_ *O* Nitrous oxide, *WASI-II* Wechsler Abbreviated Scale of Intelligence, *YSR* Youth Self Report

The entire study period will be approximately 14 weeks, including a baseline screening session, followed by phase A and phase B of the study. Specifically, phase A indicates the period in which participants will receive the N_2_O or placebo administration, and phase B refers to the 12-week follow-up period, in which participants’ mood and fluoxetine levels (fluoxetine will be administered using an open-label approach in phase B) will be monitored. All participants will required to participate in both phases A and B. Figure 1 provides a schematic illustration of the procedural flow of the study and the assessments that will be conducted in each phase.

#### Baseline screening

All participants who are referred to this study will be screened at baseline for eligibility to participate. This baseline screening session will consist of a medical review during which a thorough physical and mental health history will be taken. Additionally, participants will undergo a psychiatric review, as well as K-SADS and WASI-II for diagnostic confirmation and to determine IQ, respectively. Written consent will also be obtained during this session.

#### Phase A

On the study day, participants will be assessed by a study psychiatrist for a mental state and risk assessment. Participants will also be required to complete urine pregnancy (females) and drug screens as well as baseline mood measurements (CDRS-R, HDRS, BDI, Child Behaviour Checklist [CBCL] and Youth Self Report [YSR]). Randomisation will occur at this phase. Upon completion of all measures, the participant will be transferred to the theatre complex for N_2_O or placebo administration.

All participants will be asked to fast for 2 h for solids and fluids prior to the administration of N_2_O/placebo, as well as to adhere to routine fasting guidelines for N_2_O administration of the Department of Anaesthesia, PMH. Small sips of water will be allowed up until 30 minutes prior to the intervention. Even though N_2_O administration is standard in many dental practices and obstetrics and general ward settings, for the purposes of this study, the administration of N_2_O will be performed in the theatre complex of PMH, which allows for rapid access to emergency personnel from the theatre in the very unlikely event of complications. N_2_O or placebo will be administered by a qualified nurse or doctor via standard anaesthesia workstations using a standard anaesthesia face mask. A parent/legal guardian or a family member over the age of 18 years may accompany the patient at the theatre complex for the administration of the study treatment. The patient as well as the patient’s parent/guardian will be blinded to the group allocation.

Subjects will be randomly allocated to receive either N_2_O (50% N_2_O and 50% O_2_) or placebo (50% nitrogen/50% oxygen) over a period of 1 h in a blinded fashion. The 50% concentration of N_2_O is based on recent evidence obtained in clinical trials and is also in line with standard N_2_O treatment in the ward and clinic setting outside the operating room [3]. In line with research conducted in adults, an equal concentration of O_2_ will be used to allow adequate comparison and to minimise variability between the two conditions. A standard anaesthesia face mask for this age group will be used for treatment administration. The mask used will be connected to a standard scavenged anaesthesia machine. The total gas flow will be set at 6 L/minute, which is in line with previous research [3]. Enrolled participants will be subject to monitoring during and after treatment administration in accordance with the Australian and New Zealand College of Anaesthetists and PMH guidelines for sedation. Obtained safety data will comprise a continuous three-lead electrocardiogram, pulse oximetry, and non-invasive blood pressure and end-tidal carbon dioxide monitoring. Upon conclusion of the treatment session, subjects will be monitored for a minimum of 30 minutes and returned to the interview area. Participants will then undergo a standardised psychiatric assessment that will include an examination of mental state and risk, as well as the development of a comprehensive management plan prior to discharge.

#### Psychological assessments that will be conducted in phase A

As outlined in Fig. 1, a number of psychological assessments will be conducted in phase A, immediately pre-intervention and 2 h and 24 h after N_2_O or placebo administration. These include the BDI, HDRS, and CDRS-R. The CBCL, YSR and Clinical Global Impression (CGI) will be completed immediately pre-intervention.

#### Phase B

Phase B will start approximately 24 h after N_2_O/placebo administration, and SSRI treatment with fluoxetine (pharmacological treatment as usual [TAU]) will be initiated by the study psychiatrists. The SSRI fluoxetine will be initiated at a starting dose of 10 mg daily for 1 week and titrated up to 20 mg daily in terms of a fixed-dose open-label approach. This is in accordance with the National Institute for Health and Care Excellence clinical guideline for the treatment of depression in children and young people [20]. Fluoxetine will be continued with weekly monitoring of clinical symptoms and mood.

#### Biochemical and psychological assessments that will be conducted in phase B

Blood samples will be taken during weeks 4, 8 and 12 of phase B. These blood samples will be used to assess compliance with the fluoxetine treatment. These samples will contribute to the investigation of the relationship between mood changes and the data relating to N_2_O/placebo administration. Compliance with fluoxetine treatment will also be monitored through empty packet return and patient report. Blood samples will undergo basic laboratory processing before storage in a lockable −80 °C freezer.

Psychological assessments will be conducted on a weekly basis and will include the BDI, HDRS and CDRS. The CBCL, YSR and CGI will be completed during the weeks 6 and 12 follow-up. Measures obtained from the CBCL and the YSR during the weeks 6 and 12 follow-up will be used for additional clinical monitoring purposes. Participants are also permitted to continue their normal mental health TAU, which may include engagement in cognitive behavioural therapy or other forms of psychotherapy for depression. As per recommendation by the local ethics committee, the study does not either prescribe or restrict participation in psychological therapy. This is at the discretion of the participants and their treating clinicians. The type of psychological therapy and number of sessions over the study period will be recorded for each participant on the basis of a report from the participant’s treating clinician. Access to other treatment strategies will be accounted for in the statistical analysis of the data and will be reported upon completion of the study.

### Outcomes

The primary endpoint for the first aim of the study (to investigate the therapeutic effects of the administration of N_2_O as a single-dose inhalational anaesthetic, phase A) is the assessment of mood symptoms approximately 24 h after administration. Mood symptoms will be measured using psychometric instruments, including the BDI, HDRS and CDRS-R. Further endpoints will be mood changes after 6 weeks (secondary endpoint) and 12 weeks (tertiary endpoint). For the second and third aims of the study (to determine whether the additive effects of N_2_O and SSRI administration provide a clinically significant improvement in mood over and above the benefits from SSRI administration alone [Phase B] and to investigate N_2_O as a possible predictor of SSRI treatment response, respectively), the endpoints will be measurements of mood (indicated by the BDI, HDRS and CDRS-R) at the weeks 6 and 12 follow-up.

### Recruitment

Participants will be recruited from PMH. Referrals to the study will also be accepted from general practitioners, local paediatricians and local developmental services, as well as from Headspace clinicians (Headspace is an early intervention service for youth with mental health disorders). Participants who discontinue the study will be not be replaced.

### Blinding procedure and assignment of condition

The personnel and the location for providing N_2_O-based treatment, as well as the psychiatric team and related locations for psychiatric examinations, will be completely separated. Medical records for N_2_O/placebo administration will be kept separate from the psychiatric assessment case report forms until completion of the entire trial. Participants and their families will be blinded to the nature of the inhaled gas and will be informed that they can receive either one or the other of the two conditions during phase A. Group assignment to either N_2_O or placebo will be performed by a clinical pharmacist using a random number generator for all participants. Randomisation according to this generator will occur on a 1:1 basis with a permuted block randomisation schedule created by the PMH Pharmacy Department. Apart from the inhalational mixture, the sessions for administration of N_2_O and placebo will be indistinguishable in regard to their setting, setup and monitoring.

The order of group assignment will be kept in a secure location. Each allocation will be sealed in individual envelopes and stored in order. Individual envelopes will be opened only upon the arrival of the participant for the administration of N_2_O or placebo mixture. The only person who has access to the random group allocation will be the study nurse and/or study anaesthetist who will administer the inhalational treatment or placebo in theatre. At the end of each treatment day, the group allocation protocol and treatment supply log will be resealed and kept in a secure location. This will be the only role for the nurse/anaesthetist, who will have no contact with the psychiatric team or the patient/family throughout the study duration to ensure continuous blinding. N_2_O and medical air will be supplied by piped theatre supply from PMH (regulated by biomedical services) and not via cylinders. The nature of the gas will therefore be obscured and known only to the study nurse/anaesthetist. The study will be unblinded only in the unlikely occurrence of an adverse event requiring unblinding to ensure patient safety.

### Data preparation and statistical analysis

This trial will yield data relating to the participants’ demographics, medical history, measures of mood during phase A and phase B, and blood samples indicating fluoxetine levels during phase B of the trial. All data will be de-identified upon collection, will be entered by one designated person on the research team, and will be securely stored. Only the research team will have access to the data. Hard copies of the data and questionnaires will be kept in a locked cabinet in a locked room within a secure building.

The primary outcome measure (HDRS) will be statistically analysed using a between-subjects mixed effects linear model. Dose-response curves will also be plotted for both groups (i.e., group that received N_2_O and the group that received the placebo gas). Any differences between the two dose-response curves may highlight the difference between the interventions received in phase A. Because this is a pilot study, each group will have a maximum of 15 participants. These analyses will also be repeated for the BDI and HDRS scales. Additionally, correlational analyses (Pearson’s/Spearman’s rho, depending on data distribution) between fluoxetine levels and relevant symptom changes (HDRS and BDI) will be calculated. SPSS software (IBM, Armonk, NY, USA) will be used for these analyses.

Power analysis using G*Power showed that a sample size of 30 participants detecting effect sizes of *f* = 0.25 with an α error = 0.05 will produce power of 0.75 when using analysis of variance with repeated measures (within-between factors, number of measurements = 1, correlation among repeated measures = 0.5, non-centrality parameter λ = 7.5, critical *F* = 4.20, numerator *df* = 1, denominator *df* = 28). Whilst the power of the present study is slightly less than the conventional 0.08, it is noted that this is a pilot study, and it is anticipated that these data will inform future investigations with larger sample sizes.

### Trial management

This trial will be managed by FDZ and RMS, with assistance from JWYW and KCR. Stringent records will be kept in a master file and will include records of adverse events. In the unlikely event of an adverse event, records will be submitted by a study-specific data and safety monitoring committee, and committee members will be independent of the study team. With regard to the possible occurrence of an adverse event in phase A, N_2_O is widely used and has minimal side effects that are usually transient and vary between individuals. However, participants who experience an adverse event following the N_2_O or placebo administration will be followed in the recovery area and assessed and managed by a consultant anaesthetist until they are stable and cleared for discharge from the hospital. The chief investigator will be informed of the adverse event as soon as possible after its occurrence. All participants will be continuously monitored, and treatment with N_2_O and placebo will be conducted in a theatre with a high degree of supervision. A medical practitioner will be available at all times to assess and manage side effects if they emerge. For example, an ondansetron sublingual wafer (4–8 mg depending on weight) will be available to treat nausea and vomiting during the study period.

Stopping rules have also been developed for this trial, and the development of these rules has been done taking into consideration common and serious adverse events which can occur in adolescents being treated for a major depressive episode with antidepressant medication. Because it may be difficult to disentangle whether an adverse event is due to treatment or part of the depressive disorder, this study will be halted and reviewed if the following number of adverse events occur: an event of a completed suicide, two events of attempted suicide, four individual reports of an increase in non-suicidal deliberate self-harm, three individuals reporting the development of hypomania or two patients reporting the development of mania, and one report of severe psychotic symptoms requiring hospitalisation or intensive community-based treatment. The rates of the following possible adverse events have been estimated from the Treatment of Adolescent Depression Study [21], because it has published results based on the largest available cohort of comparable patients. If patient symptoms worsen during the trial, they may be excluded from the study and will not be replaced. However, they will receive a care plan, and the study doctors will facilitate access to community-based mental health care.

## Discussion

Findings of the proposed project have the potential for both an immediate and lasting impact. Given the very large effect sizes found in the administration of inhaled N_2_O in the adult cohort [3], we expect the administration of N_2_O in an adolescent cohort to yield similar results. Short-term impacts can include the alleviation of depressive symptoms for participants within 24 h [3], and depending on their duration, further research will need to determine how to overcome the time until the onset of the observable clinical benefits of SSRIs (in the present case, fluoxetine). Such findings could be of great impact because currently some children and adolescents with depressive symptoms may not experience any symptom alleviation up to several weeks following the initiation of SSRIs. Further, such effects would be beneficial to both the children and their families and therefore allow the children to re-engage with typical daily activities at an earlier point in time. This is in line with national mental health reviews that have advocated for the early intervention in patients with mental health disorders [22]. Additionally, earlier re-engagement may have far-reaching and long-term benefits because it may minimise the disruption to children’s developmental trajectories.

As with any new research findings, if the present study were to show some first beneficial effects of N_2_O administration, such findings would need replication. In addition to the proposed immediate and lasting clinical impacts for children and adolescents, there is potential for this novel therapy to move from the bench to the bedside to inform both clinical practice and service delivery. In addition to the mental health setting, the possible immediate reduction in depressive symptoms following N_2_O administration may have application in general or specialist health settings where depressed children and adolescents may present (e.g., theatre) or have long lengths of stay as inpatients (e.g., rehabilitation). There could also be merit in pursuing a more translational research perspective by focusing on the design, training, financing, and policy and procedural requirements of this promising intervention. This is made even more possible by increased funding opportunities for this type of research. Although a lot of clinical research does not find a way into practice (e.g., [23, 24]), there now exist many knowledge translation and implementation frameworks, theories and models, and an accompanying literature of applied research which can guide the researcher, clinician, policy maker and/or educator to ensure that this potential new treatment option becomes standard practice for improving mental health in children and adolescents.

### Trial status

This article describes protocol version 8, dated 17 November 2016, and was prepared with reference to the Standard Protocol Items: Recommendations for Interventional Trials (SPIRIT) checklist please see Additional file 1. Recruitment stated in October 2017.

## Additional file

Additional file 1: SPIRIT 2013 checklist: recommended items to address in a clinical trial protocol and related documents. (DOC 120 kb)

## Abbreviations

BDI: Beck Depression Inventory
CBCL: Child Behaviour Checklist
CDRS-R: Children’s Depression Rating Scale–Revised
CGI: Clinical Global Impression
ECG: Electrocardiography
HDRS: Hamilton Depression Rating Scale
5-HT: Serotonin
K-SADS: Kiddie Schedule for Affective Disorders and Schizophrenia
NMDA: *N*-methyl-d-aspartate
N_2_O: Nitrous oxide
NOTAD: Nitrous oxide (N_2_O) and subsequent open-label SSRI treatment of adolescents with depression study
PMHF: Princess Margaret Hospital Foundation
SPIRIT: Standard Protocol Items: Recommendations for Interventional Trials
SSRI: Selective serotonin reuptake inhibitor
TAU: Treatment as usual
TRD: Treatment-resistant depression
WASI-II: Wechsler Abbreviated Scale of Intelligence
YSR: Youth Self Report
